# Concomitant Pyoderma Gangrenosum-like and Amicrobial Pustulosis of the Folds: a Case Report

**DOI:** 10.1007/s10875-020-00819-1

**Published:** 2020-07-09

**Authors:** Paola Facheris, Maria De Santis, Luigi Gargiulo, Giulia Pavia, Mario Valenti, Sofia Manara, Riccardo G. Borroni, Antonio Costanzo, Alessandra Narcisi

**Affiliations:** 1 Department of Dermatology, Humanitas Clinical and Research Center-IRCCS, Via Manzoni 56, 20089 Rozzano, MI Italy; 2 Department of Biomedical Sciences, Humanitas University, Pieve Emanuele, MI Italy; 3 Department of Rheumatology and Clinical Immunology, Humanitas Clinical and Research Center-IRCCS, Rozzano, MI Italy; 4 Department of Pathology, Humanitas Clinical and Research Center-IRCCS, Rozzano, MI Italy

## Introduction

To the Editor,

The skin is one of the most commonly affected organs by the extraintestinal manifestations of inflammatory bowel disease (IBD), including Crohn’s disease (CD) and ulcerative colitis (UC). UC may present cutaneous manifestation in 5% to 11% of patients [1]. Based on their pathophysiology, cutaneous manifestations associated with IBD can be classified in 4 categories as follows: specific, reactive, associated, and induced by IBD treatment [1]. Reactive cutaneous manifestations have a different histopathology but have close physiopathologic link with the intestinal disease, being autoinflammatory skin diseases such as neutrophilic dermatoses.

## Case Presentation

An 18-year-old patient presented to our dermatologic outpatient clinic for the appearance of an ulcer on the lateral side of her right ankle. Her past medical history was notable for spondylarthritis and ulcerative colitis since the age of 12, previously treated with two TNF-alfa inhibitors (infliximab and adalimumab). At the time of our evaluation, the patient was under treatment with low doses of systemic steroids following a recent exacerbation of the intestinal disease. The cutaneous lesion had been already evaluated elsewhere a week before the presentation at our department and a cultural exam resulted completely sterile. However, the patient complained of a progressive worsening of the ulcer. On physical examination, there was the presence of a 5-cm tender ulcer exhibiting violaceous and undermined borders with a purulent base. Intense erythema was noted around the borders of the ulcers. In order to exclude the suspect of osteomyelitis, a CT scan and an MRI of the left foot and leg were performed. Both exams gave negative results concerning osteomyelitis or abscess formation but highlighted a significant involvement of the subcutaneous tissue. Also, numerous pustules were noted at the inguinal folds bilaterally and the patient referred similar episodes in the past treated as folliculitis with topical antibiotic therapy.

The patient was admitted for further evaluation and during hospitalization other lesions appeared as follows: pustules were present on the left thigh, right thigh, and left gluteus. In a few hours, the pustules coalesced forming ulcers with violaceous undermined borders and a necrotic base. The patient had developed a total of four pyoderma gangrenosum-like lesions (right ankle, left thigh, right thigh, and left gluteus) and numerous other pustules located at the inguinal folds clinically compatible with amicrobial pustulosis of the folds.

**Fig. 1:**
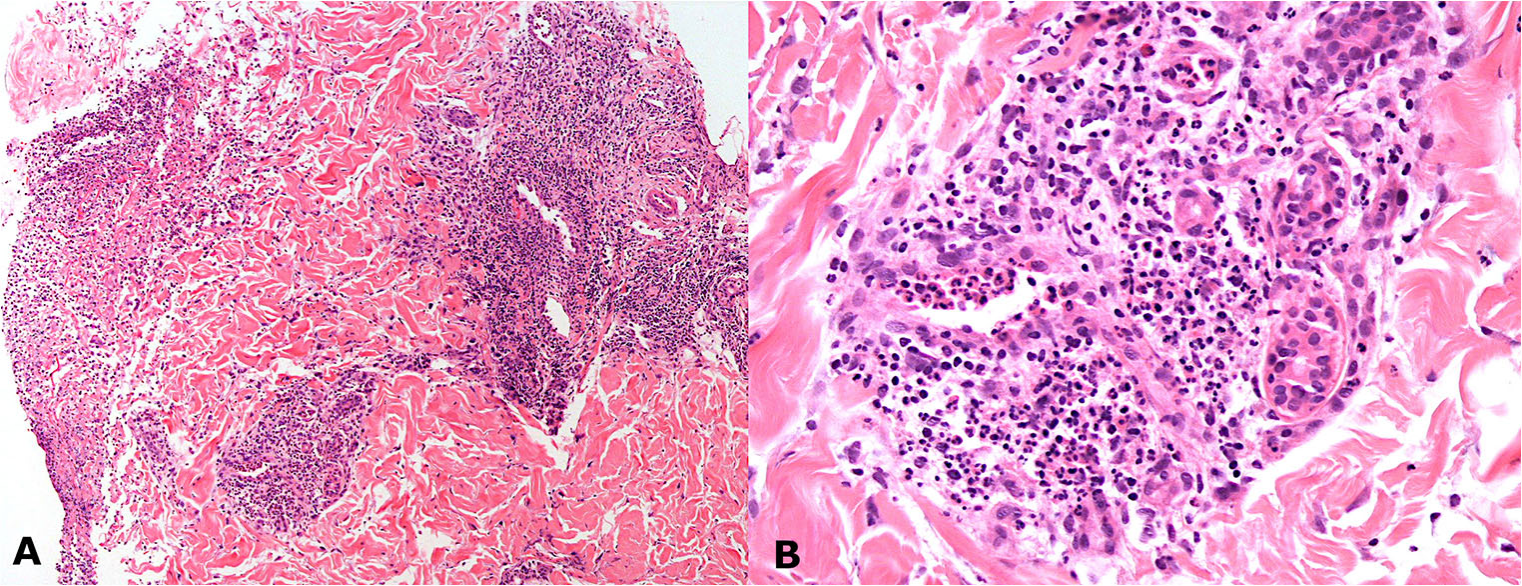
Histopathological images of the skin biopsy of one pustule of the left inguinal fold. Perivascular and periadnexal intense mixed inflammatory infiltrate rich in neutrophils, absence of eosinophils, and no signs of microvasculitis (Fig. 2a: × 10 magnification, Fig. 2b: × 40 magnification). These findings make the diagnosis compatible with a form of neutrophilic dermatosis

Severe tenderness of the pyoderma gangrenous-like lesions combined with a severe psychological and physical distress did not allow a skin biopsy of the pyoderma gangrenosum-like ulcers. However, the patient agreed to the skin biopsy of one of the pustules of the left inguinal fold and the histopathology report was consisted with a neutrophilic dermatosis showing perivascular and periadnexal intense mixed inflammatory infiltrate rich in neutrophils. Absence of eosinophils and no signs of microvasculitis were reported (Fig. 1). A cutaneous swab from the same lesions gave negative cultural results and was reported as completely sterile. The serological panel of the patient was as follows: negative HLA-B27, ANA 1:160 homogenous (absent anti-DNA and anti-ENA), positive pANCA (anti-MPO), and negative cANCA (Anti-Pr3). A colonoscopy showed intense disease activity with the presence of many pseudopolyps. Based on these findings and on the clinical and histopathological characteristics, the pustular lesions of the inguinal folds can be considered very similar to amicrobial pustulosis of the folds [2].

**Fig. 2:**
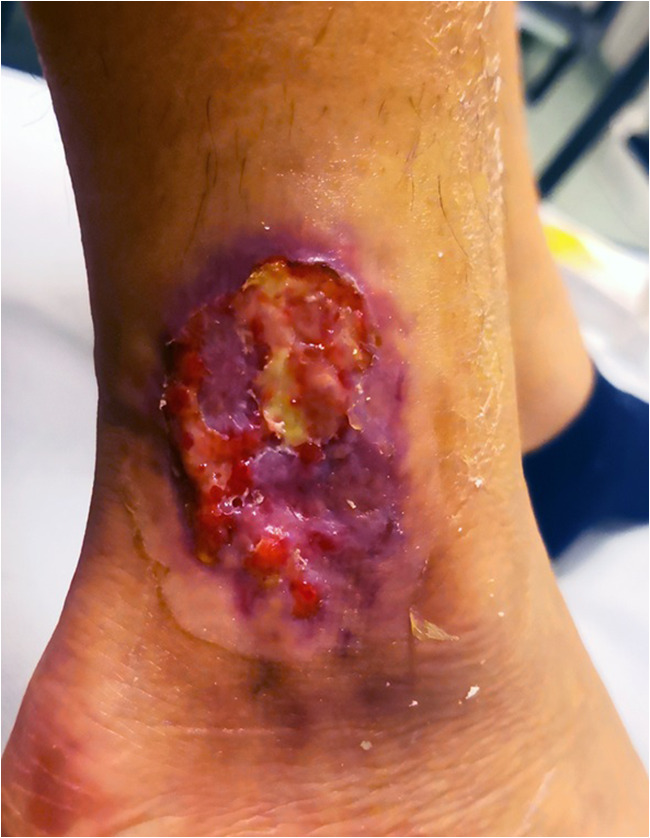
Pyoderma gangrenosum of the left ankle after 10 days of therapy. The lesion is gradually improving: the central necrotic area is reducing, and re-epithelization with scarring is starting

Endovenous cyclosporine at a dose of 3 mg/kg/day aiming a serum concentration of 100–200 ng/ml and local medications with topical tacrolimus ointment 0.1% were started. Progressive improvement of both the intestinal and skin diseases was rapid: The PG-like lesions gradually improved (Fig. 2) and the pustules disappeared by the time of discharge, 13 days after the admission. The patient continued the treatment with oral cyclosporine at a dose of 150 mg/day (3 mg/kg/day) for 3 months after the discharge. The dosage of oral cyclosporine was progressively tapered until achieving suspension in 6 months.

## Discussion

Neutrophilic dermatoses represent a clinically heterogeneous group of disorders whose hallmark is the presence of neutrophilic infiltrate in the skin and rarely in internal organs.

Pyoderma gangrenosum (PG) is a rare inflammatory neutrophilic dermatosis that typically presents as one or more painful ulcers with violaceous, undermined borders on the lower extremities. Less common presentations include tender nodules or pustules on other sites of the body. PG is the second most frequently documented cutaneous manifestation in patients with IBD after erythema nodosum [1]. It affects equally men and women, with a peak incidence between 20 and 50 years [1]. The temporal relationship between IBD and PG is still unclear. One study on 14 patients with UC demonstrated no temporal association between the bowel exacerbations and the course of PG lesions [3]; whereas another study on 34 IBD patients found that PG was commonly diagnosed when the underlying IBD was active [4]. In the case of our patient, it was not possible to confirm the clinical diagnosis with a biopsy, which represents the major diagnostic criteria stated by the Delphi diagnostic criteria [5]. However, the clinical presentation and course, the rapid response to immunosuppressive therapy and the wound healing with scarring are all factors that make our case to fulfill almost all the minor criteria and make the diagnosis of pyoderma gangrenosum highly probable.

Amicrobial pustulosis of the folds (AFP) is a rare, chronic, relapsing dermatosis that affects almost exclusively young women with sudden onset of follicular and non-follicular sterile pustular lesions involving the main cutaneous folds, anogenital area, and scalp as well as minor skin folds, particularly the area around the nostrils, retroauricular regions, and external auditory canals.[3] The histology is characterized by subcorneal pustules associated with a mainly neutrophilic infiltrate in the dermis, which has brought many authors to include APF in the spectrum of neutrophilic dermatoses [2]. APF has been reported to be associated with autoimmune diseases, including lupus erythematosus, myasthenia gravis, mixed connective tissue disease, Sjögren’s syndrome, idiopathic thrombocytopenic purpura, and immunoglobulin A nephropathy [2]. In 2011, Lee et al. described the first case of APF associated with Crohn’s disease [6]. In the case of our patient, the clinical manifestation and the biopsy report are highly suggestive for amicrobial pustulosis of the folds. Based on the clinical history reported by the patient, it is probable that the previous episodes of folliculitis were instead episodes of APF. These episodes were also reported to be simultaneous to UC worsening.

We believe that the concomitant appearance of multiple pyoderma gangrenosum-like lesions and amicrobial pustulosis of the folds suggest that these two entities are manifestations of a continuous spectrum in the context of neutrophilic dermatoses associated with IBD.

## Conclusions

While pyoderma gangrenosum is a clinically well-characterized entity, APF is a rarer disease whose clinical presentation may lead to misdiagnosis as happened in the case of our patient. In fact, the patient reported previous episodes of APF misdiagnosed as folliculitis resistant to antibiotic treatment. Pustular manifestations can be a sign of neutrophilic dermatoses as represented in this case.

The simultaneous clinical presentation of pyoderma gangrenosum lesions and amicrobial pustulosis of the folds make the case of our patient a unique example of the important relationship of neutrophilic dermatoses with IBD, in this case UC. PG and APF may be linked by common pathogenetic mechanisms that led to the onset of both diseases in the context of a UC flare. PG and its syndromic forms can be considered as manifestations of a single clinic-pathological spectrum similar to that of autoinflammatory diseases, suggesting common physiopathological mechanisms.

In the case of our patient, intravenous cyclosporine demonstrated to be an efficacious treatment for both the cutaneous lesions PG-like and APF and the intestinal disease.
